# A One-Year Retrospective Study of the Occurrence of Sport-Related Concussions in Japanese University Sports: Characteristics of Athletes with Multiple Occurrences

**DOI:** 10.3390/sports14050175

**Published:** 2026-04-28

**Authors:** Yuki Muramoto, Takeshi Kimura, Akira Kinoda, Yoshinori Katsumata, Kazuki Sato

**Affiliations:** 1 Institute for Integrated Sports Medicine, Keio University School of Medicine, 35 Shinanomachi, Shinjuku-ku, Tokyo 160-8582, Japan; yukimuramoto1019@gmail.com (Y.M.);; 2 Department of Cardiology, Keio University School of Medicine, 35 Shinanomachi, Shinjuku-ku, Tokyo 160-8582, Japan

**Keywords:** Japan Association for University Athletics and Sport, sport-related concussion, web-based injury survey, body mass index, epidemiology

## Abstract

**Background/Objectives:** This study aimed to investigate the occurrence of sport-related concussions (SRCs) in Japanese university sports and to identify factors associated with experiencing multiple SRCs within a single season. **Methods:** Data were collected through a web-based survey conducted from June to October 2022, focusing on injuries sustained between April 2021 and March 2022. Participants were recruited from universities affiliated with the Japan Association for University Athletics and Sport and were required to be actively participating in sports. **Results:** Overall, 10,978 participants were analyzed; 195 reported SRCs, whereas 10,783 did not. Athletes who reported SRCs were significantly taller and heavier, had a higher body mass index, and included a higher proportion of male athletes than those who did not report SRCs. SRCs were most frequently reported in rugby football, American football, soccer, and lacrosse. Among athletes with SRCs, those with multiple SRCs tended to be taller and were significantly heavier. They also had a significantly higher proportion of severe first SRCs during the season. **Conclusions:** SRCs were most frequently reported in rugby football, American football, soccer, and lacrosse, and greater severity of the initial SRC in a season was associated with multiple SRC occurrence. These findings should be interpreted with caution because SRCs were self-reported and not clinically verified, mild cases may have been underreported, and time-loss-based severity may not reflect clinical severity.

## 1. Introduction

Sport-related concussions (SRCs) are an important issue in sports medicine because they can occur in a wide range of sports. Previous studies have reported relatively high concussion rates in sports such as American football, rugby, and ice hockey, and a substantial burden has also been reported in women’s soccer [1,2,3,4,5,6,7,8,9]. In addition, the pattern of SRC occurrence may vary not only according to sport characteristics but also according to playing position. For example, differences in concussion incidence have been reported between backs and forwards in rugby, and some studies in women’s soccer have reported a higher frequency among forwards and defenders [8,9]. These findings suggest that SRC occurrence may be influenced by sport-specific characteristics, playing position, and differences in contact exposure. While most athletes recover within a short period of time, some experience prolonged symptoms, particularly those who experience multiple SRCs within a single season. Repeated SRCs have been associated with prolonged recovery times and an increased risk of long-term cognitive and neurological issues, such as chronic traumatic encephalopathy [10,11,12]. Previous studies suggest that factors such as body size, playing position, and sport type may influence the likelihood of sustaining multiple SRCs [13,14]. However, few studies have systematically examined these factors in university athletes.

Identifying the risk factors associated with multiple SRCs is crucial for the development of effective prevention strategies. Therefore, this study aimed to investigate the occurrence of multiple SRCs and identify the factors associated with their increased incidence among Japanese university athletes.

## 2. Methods

### 2.1. Research Design

In this cross-sectional observational study, data were collected using a web-based survey conducted between June and October 2022. The Keio University School of Medicine Ethics Committee approved the study protocol (approval number: 20211158; approved on 29 March 2022). All participants provided informed consent before participating in the study. The study was conducted in accordance with the Declaration of Helsinki and followed the guidelines of the Strengthening the Reporting of Observational Studies in Epidemiology Extension for Sports Injury and Illness Surveillance consensus statement [15,16].

### 2.2. Athlete Recruitment

**Figure 1:**
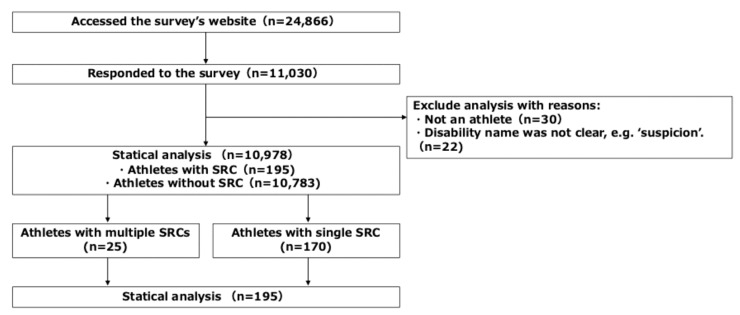
Flow diagram of participant selection. SRC, sport-related concussion. Athletes with SRC reported at least one SRC during the study season. Athletes with multiple SRCs reported two or more SRCs within a single season, whereas athletes with a single SRC reported one SRC.

Questionnaires were sent to coaches in all competitions at 225 universities affiliated with the Japan Association for University Athletics and Sport. The coaches were asked to distribute the survey to all athletes at the time of the survey. The athletes were required to provide consent before participating in the survey. The inclusion criterion was active participation in athletic activities at the time of the survey. A total of 24,866 individuals were approached to participate in this study. Of these, 11,030 participants provided a complete response. Thirty respondents were excluded because they were not athletes, and 22 were excluded because of unclear diagnoses, such as “suspected” conditions, resulting in a final analytic sample of 10,978 participants. Among these, 195 athletes self-reported at least one SRC during the April 2021–March 2022 season and were classified as the SRC group, whereas 10,783 reported no SRC and were classified as the no-SRC group. Among athletes with SRC, those who reported two or more SRCs within a single season were classified as the multiple-SRC group, whereas those who reported one SRC only were classified as the single-SRC group. The participant selection process is illustrated in Figure 1.

### 2.3. Survey Questions

The survey was administered through a dedicated website created specifically for this study and was self-reported by the athletes. The survey questions were derived from the Japanese Society of Clinical Sports Medicine and modified to suit collegiate athletes [17,18]. The questionnaire was validated using the Delphi method, which is well agreed upon by experts in sports science [13]. The survey collected information on athlete characteristics (age, height, and weight), type of sport, and injuries sustained in the past year (determined by posing the question: “Have you had any injuries in the last year [April 2021–March 2022] in your main sport?”). For the three most serious injuries reported, follow-up questions were posed to gather specific details, including injury location, diagnosis, duration of absence from sports participation, and time loss-based injury severity. Injury severity was determined based on the duration of absence from training or competition, and was classified using a modified Bahr-Bowen approach [18]. This severity classification was based on time loss from sport participation and was not determined based on neurological symptoms, loss of consciousness, or other clinical indicators. Injury severity was rated as no absence (no absence), mild (absence for 1 day to 1 week), moderate (absence for 1 week to 1 month), and severe (absence for >1 month) [18].

### 2.4. Data Analysis

IBM SPSS Statistics (IBM Corp., Armonk, NY, USA; version 28.0) was used for the descriptive and univariable analyses. Qualitative and quantitative data are presented as frequencies, means, and standard deviations. Statistical analysis of quantitative data was performed using an unpaired *t*-test. Fisher’s exact test was used to analyze qualitative data. Cohen’s d and phi (φ) coefficients were calculated to measure the effect size. Statistical significance was set at *p* < 0.05. Given the small size of the multiple SRC group (*n* = 25), Mann–Whitney U tests were additionally performed as a sensitivity analysis for continuous variables. To identify factors independently associated with multiple SRCs, Firth penalized logistic regression was conducted among athletes who experienced at least one SRC (*n* = 195), with multiple SRC occurrence (yes/no) as the outcome. Firth’s penalization was applied to reduce small-sample bias given the limited number of athletes with multiple SRCs (*n* = 25). The model included the SRC severity of the first event in the season, sex, and contact sports participation as covariates. Results are reported as odds ratios (ORs) with 95% confidence intervals (CIs).

## 3. Results

Table 1 shows the participants’ background characteristics. The SRC group had a mean height, weight, and body mass index (BMI) of 171.0 ± 8.0 cm, 79.1 ± 18.1 kg, and 26.8 ± 5.1 kg/m^2^, respectively. The corresponding values in the non-SRC group were 168.0 ± 8.9 cm, 66.0 ± 13.9 kg, and 23.2 ± 3.6 kg/m^2^. The SRC group was significantly taller (mean difference = 2.94 cm, *p* < 0.01, Cohen’s d = 0.33) and heavier (mean difference = 13.29 kg, *p* < 0.001, Cohen’s d = 1.13) and had a higher BMI (mean difference = 3.7 kg/m^2^, *p* < 0.001, Cohen’s d = 1.02). Furthermore, in sports with more than 10 SRC cases, such as rugby football, American football, lacrosse, and soccer, participants in the SRC group who played rugby football and American football had a higher BMI (Supplementary Table S1). The SRC group also had a significantly higher proportion of male participants (*p* < 0.01, φ = 0.06).

### 3.1. Distribution of SRCs by Sport and Severity

**Figure 2:**
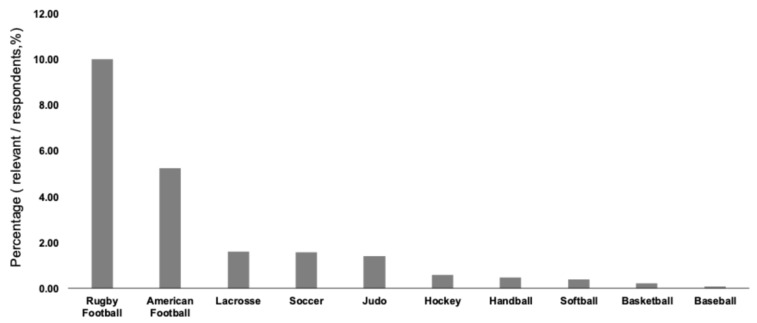
Percentage of sport-related concussions in competitions with more than 100 responses.

Table 2 summarizes the distribution of athletes with SRCs according to sports and injury severity. Rugby football had the highest occurrence, accounting for 42.6% of the athletes who experienced SRCs, followed by American football (24.1%) and lacrosse (13.8%). Figure 2 shows the proportion of SRC cases relative to the total number of respondents for sports, with more than 100 responses. Similarly, contact-intensive sports, such as rugby football, American football, and lacrosse, showed higher SRC proportions.

SRC severity was classified based on the number of days athletes were unable to participate in sports because of injury. In more than half (56.4%) of the cases, SRC severity was moderate, requiring 8–31 days of restricted activity. Severe SRC cases requiring ≥32 days of restricted activity accounted for 16.4% of the cases. Mild SRC cases (1–7 days of restricted activity) and non-time-loss SRC cases (0 days) accounted for 23.6% and 3.6% of the cases, respectively.

### 3.2. Comparison Between Athletes Who Did and Did Not Experience Multiple SRCs

Twenty-five athletes self-reported experiencing an SRC two or more times in a single season (April 2021–March 2022); they were categorized as the multiple SRC group, whereas 170 athletes who reported experiencing an SRC only once were defined as the non-multiple SRC group (Figure 1).

Table 3 presents a comparison between the multiple and non-multiple SRC groups. Athletes who reported multiple SRCs tended to be taller (mean difference = 2.94 cm, Cohen’s d = 0.37, *p* = 0.086) and significantly heavier (mean difference = 8.29 kg, Cohen’s d = 0.46, *p* = 0.032). There was no significant difference in BMI between the multiple and non-multiple SRC groups (mean difference = 1.99 kg/m^2^, Cohen’s d = 0.40, *p* = 0.066).

Regarding SRC severity, the multiple SRC group had a significantly higher proportion of severe cases during the first SRC of the season (44.0% vs. 12.4%, *p* < 0.01, φ = 0.29). When comparing sports types, no significant differences were observed between the multiple SRC and non-multiple SRC groups.

To further examine whether the severity of the first SRC was independently associated with multiple SRC occurrence, Firth penalized logistic regression was conducted (Table 4). After adjusting for sex and contact sport participation, the severity of the first SRC remained significantly associated with multiple SRC occurrence (OR = 2.23, 95% CI: 1.31–3.80, *p* = 0.003). Neither sex nor contact sport participation was independently associated with the outcome. These findings suggest that greater severity of the initial SRC was associated with multiple SRC occurrence within a season, independent of sex and contact sport participation.

## 4. Discussion

This study investigated the occurrence of SRCs in university sports and examined the factors associated with experiencing multiple SRCs within a single season. The results indicated that SRCs were more frequently reported among athletes with larger physiques in rugby football and American football. In addition, multiple SRCs within the same season were more commonly observed among athletes whose first SRC in the season was classified as more severe.

### 4.1. Interpretation of Sport-Specific SRC Occurrence and Severity

The study results align with previous findings, indicating that the incidence rates of SRCs were high in male athletes, athletes with a high BMI, and rugby football and American football players [19,20,21]. These findings should also be interpreted in the context of SRC epidemiology among Japanese collegiate athletes. Previous studies in Japanese collegiate athletes have shown that SRC burden and SRC history are greater in contact sports than in limited-contact and noncontact sports, and that sport type and sex may influence SRC epidemiology and reporting behaviors [19,22]. Therefore, the high SRC frequency observed in rugby football and American football in the present study is broadly consistent with previous reports in Japanese collegiate athletes. However, BMI itself may not be a direct causal risk factor; rather, particularly in rugby football and American football, it may reflect sport type, playing position, and cumulative collision exposure [23,24,25,26]. In sports such as lacrosse and soccer, which also show a high incidence of SRCs, no association with BMI was observed. The SRC risk in lacrosse and soccer may stem not only from player-to-player contact but also from head impacts due to balls or falls [27,28]. Therefore, the relationship between body size and SRC may differ according to the sport. However, because our survey did not directly assess playing position, contact exposure, or the specific mechanism of injury, these interpretations should be interpreted with caution.

### 4.2. Comparison of Physical Size and SRC Severity Between the Multiple SRC and Non-Multiple SRC Groups

This study revealed that athletes who experienced multiple SRCs within a single season tended to be taller and significantly heavier. In rugby and American football, particularly for forward players, frequent and intense contact is an inherent part of the game, leading to greater exposure to high-impact collisions [23,24]. Therefore, athletes with larger physiques may have greater exposure to contact situations associated with multiple SRCs [23,24].

In this study, multiple SRCs were operationally defined as two or more SRCs occurring within a single season. Although there is no universally standardized definition, this approach is conceptually consistent with prior studies examining same-year or same-season repeat concussions [29]. Risk factors for multiple SRCs include a history of SRCs [27], return to play without adequate recovery [28], and participation in contact sports [20]. These factors may interact with physical attributes. For instance, larger athletes are often assigned to positions with frequent contact, which increases their exposure to head impact. Additionally, returning to play without sufficient recovery may result in residual brain damage, making athletes more susceptible to subsequent SRCs.

However, in this study, multiple SRCs within the same season were more frequently reported among athletes whose first SRC of the season was classified as severe and involved a longer time loss from sports participation. This result differs from the widely accepted notion that return to premature play increases the risk of recurrence. Recent studies suggest that a prolonged recovery period may also contribute to an increased risk of recurrent concussions [30,31,32,33], highlighting the need for further investigation.

Since this study did not collect detailed information on prior concussion history or the severity of the initial SRC, the impact of appropriate return-to-play timing on preventing multiple SRCs remains unclear. Future research is needed to determine how recovery duration influences SRC recurrence and to establish evidence-based guidelines for concussion management.

## 5. Limitations

This study has several limitations that should be considered when interpreting the results. First, the response rate was unknown because it was unclear how many athletes completed the questionnaire. Additionally, while the study included athletes currently participating in sports, athletes undergoing rehabilitation or those who missed the season because of severe injuries may not have responded. Second, the retrospective nature of injury reporting may have been subject to recall and cognitive bias. This is particularly relevant if a significant amount of time has passed since the injury occurred, which could affect the accuracy of self-reported data, such as injury severity. Furthermore, our methodology only collected follow-up information on the three most severe injuries reported by each athlete. As a result, mild SRCs or repeated concussions that were not perceived as the most severe injuries may have been underreported, potentially leading to an underestimation of multiple SRC incidence. In addition, detailed information on prior concussion history was not collected. Therefore, prior concussion history may have acted as a confounding factor, potentially influencing both the severity of the initial SRC and the likelihood of recurrence. Future studies should consider collecting comprehensive injury histories to improve accuracy. Third, to obtain accurate injury incidence rates, the number of injuries per 1000 athlete exposures or per 1000 athlete hours in games or training sessions must be calculated [15]. However, because records of training volume, training hours, number of matches, and actual opportunities for contact exposure were not available, exposure-adjusted incidence rates could not be calculated in this study. Therefore, the sport-specific findings in this study should be interpreted as descriptive distributions of reported SRC cases rather than as exposure-adjusted estimates of the true risk. Fourth, the number of athletes in the multiple SRC group was small (*n* = 25), which may have limited the statistical power and affected the precision and stability of the estimated associations. Although the Firth penalized logistic regression was used to reduce small-sample bias in the multivariate analysis, the results should still be interpreted with caution. Fifth, the data were self-reported by the athletes, and specific diagnoses were not verified. In addition, injury severity in this study was classified based on self-reported time loss from sport participation, which may have been influenced by external factors such as team schedules, return-to-play protocols, and sport-specific characteristics, and therefore may not fully reflect neurological severity. In contrast, in the National Collegiate Athletics Association survey methodology, injuries are assessed and recorded by physicians or certified athletic trainers [34]. Additionally, variables such as height, weight, and BMI were self-reported, which may have introduced measurement errors and potential bias. While our findings suggest a relationship between physical characteristics and SRC risk, future research should incorporate direct measurements and medical assessments to validate these associations more rigorously. Finally, it is challenging to clarify the specific circumstances leading to these injuries. Future research should focus on identifying the sources of injuries to effectively promote awareness and preventive measures.

## 6. Conclusions

This study suggests that, in contact sports such as rugby football and American football, athletes with higher BMI, height, and weight experienced more SRCs. In contrast, BMI was not associated with SRC frequency in sports such as lacrosse and soccer, suggesting that the mechanisms of SRC occurrence vary among sports. Furthermore, athletes who sustained multiple SRCs within a single season were significantly heavier and experienced a more severe first SRC of the season. These findings should be interpreted with caution because the SRCs were self-reported and not clinically verified.

## Figures and Tables

**Table 1:** Characteristics of participants (continuous variables: mean (SD), categories: *n* (%)).

	All (*n* = 10,978)	Athlete with SRC (*n* = 195)	Athlete Without SRC (*n* = 10,783)
Height (cm)	168.0 (8.6)	**171.0 (8.0)** *	168.0 (8.9)
Mass (kg)	66.2 (14.1)	**79.1 (18.1)** *	66.0 (13.9)
BMI (kg/m^2^)	23.2 (3.6)	**26.8 (5.1)** *	23.2 (3.6)
Age (year)	19.9 (1.3)	20.3 (1.2)	19.9 (1.3)
Sex			
Male, *n* (%)	6829 (62.2)	**166 (85.1)** *	6663 (61.8)
Female	4093 (37.3)	29 (14.8)	4064 (37.7)
Unspecified	56 (0.5)	0	56 (0.5)
Year in School			
1	3624 (33.0)	26 (13.3)	3598 (33.4)
2	2786 (25.4)	55 (28.2)	2731 (25.3)
3	2342 (21.3)	56 (28.7)	2286 (21.2)
4	2141 (19.5)	55 (28.2)	2086 (19.3)
graduated	85 (0.8)	3 (1.6)	82 (0.8)

* *p* < 0.05; significant differences between conditions with and without sport-related concussion. SRC; Sport-related concussion, BMI; Body mass index.

**Table 2:** Competitions in which sport-related concussion occurred, severity (category: *n* (%)).

Sports	*n* (%)
Rugby Football	83 (42.6%)
American Football	47 (24.1%)
Lacrosse	27 (13.8%)
Soccer	14 (7.2%)
Judo	8 (4.1%)
Softball	5 (2.6%)
Cheerleading	3 (1.5%)
Hockey	1 (0.5%)
Handball	1 (0.5%)
Basketball	1 (0.5%)
Baseball	1 (0.5%)
Wrestling	1 (0.5%)
Shorinji Kempo	1 (0.5%)
Aikido	1 (0.5%)
Cycling	1 (0.5%)
Severity	
Non time loss (0 day)	7 (3.6%)
Mild (1–7 days)	46 (23.6%)
Moderate (8–31 days)	110 (56.4%)
Severe (≥32 days)	32 (16.4%)

**Table 3:** Comparison of participants with and without multiple sport-related concussion (continuous variables: mean (SD), categories: *n* (%)).

	Athletes with Multiple SRCs (*n* = 25)	Athletes with a Single SRC (*n* = 170)
Height (cm)	173.5 (7.8)	171.0 (8.0)
Mass (kg)	**86.3 (19.8)** *	78.0 (17.6)
BMI (kg/m^2^)	28.6 (6.5)	26.6 (4.8)
Age (year)	20.2 (1.5)	20.4 (1.2)
Sex		
Male, *n* (%)	23 (92.0)	143 (84.1)
Female	2 (8.0)	27 (15.9)
Severity		
Non time loss (0 day)	1 (4.0)	6 (3.5)
Mild (1–7 days)	3 (12.0)	43 (25.3)
Moderate (8–31 days)	10 (40.0)	100 (58.8)
Severe (≥32 days)	**11 (44.0)** *	21 (12.4)
Sports		
Rugby Football	12 (48.0%)	71 (41.8%)
American Football	7 (28.0%)	40 (23.5%)
Lacrosse	3 (12.0%)	24 (14.1%)
Soccer	1 (4.0%)	13 (7.6%)
Judo	1 (4.0%)	7 (4.1%)
Softball	1 (4.0%)	4 (2.4%)
Cheerleading	0 (0.0%)	3 (1.8%)
Hockey	0 (0.0%)	1 (0.6%)
Handball	0 (0.0%)	1 (0.6%)
Basketball	0 (0.0%)	1 (0.6%)
Baseball	0 (0.0%)	1 (0.6%)
Wrestling	0 (0.0%)	1 (0.6%)
Shorinji Kempo	0 (0.0%)	1 (0.6%)
Aikido	0 (0.0%)	1 (0.6%)
Cycling	0 (0.0%)	1 (0.6%)

* *p* < 0.05; significant differences between athletes with multiple SRCs and those with a single SRC. SRC; Sport-related concussion, BMI; Body mass index.

**Table 4:** Firth penalized logistic regression for factors associated with multiple sport-related concussions (*n* = 25 events among 195 athletes with at least one SRC).

Variable	OR	95% CI	*p*-Value
SRC severity (per 1-level increase)	2.23	(1.31–3.80)	**0.003**
Male sex	1.71	(0.39–7.49)	0.478
Contact sport	0.74	(0.10–5.47)	0.770

OR, odds ratio; CI, confidence interval; SRC, sport-related concussion. Firth penalized logistic regression was applied to reduce small-sample bias, given the limited number of athletes with multiple SRCs. SRC severity was modeled as an ordinal variable according to the duration of absence from sport: no absence, 1–7 days, 8–31 days, and ≥32 days.

## Data Availability

All data from this study are presented in this manuscript or are available from the corresponding author upon reasonable request. Source data are provided in this study.

## Supplementary Materials

The following supporting information can be downloaded at: https://www.mdpi.com/article/10.3390/sports14050175/s1, Supplementary Table S1: Comparison of physical characteristics between athletes with and without sport-related concussion by sport.

## Abbreviations

The following abbreviations are used in this manuscript:

BMI	Body mass index
CI	Confidence intervals
OR	Odds ratios
SRC	Sport-related concussion

## References

[1] Patricios J.S., Schneider K.J., Dvorak J., Ahmed O.H., Blauwet C., Cantu R.C., Davis G.A., Echemendia R.J., Makdissi M., McNamee M. (2023). Consensus statement on concussion in sport: The 6th International Conference on Concussion in Sport-Amsterdam, October 2022. Br. J. Sports Med..

[2] Graham R., Rivara F.P., Ford M.A. (2014). Committee on Sports-Related Concussions in Youth. Board on Children, Youth, and Families; Institute of Medicine. Sports-Related Concussions in Youth: Improving the Science, Changing the Culture.

[3] Marshall C.M. (2012). Sports-related concussion: A narrative review of the literature. J. Can. Chiropr. Assoc..

[4] Kerr Z.Y., Zuckerman S.L., Wasserman E.B., Covassin T., Djoko A., Dompier T.P. (2016). Concussion symptoms and return to play time in youth, high school, and college American football athletes. JAMA Pediatr..

[5] McCrory P., Meeuwisse W., Dvořák J., Aubry M., Bailes J., Broglio S., Cantu R.C., Cassidy D., Echemendia R.J., Castellani R.J. (2017). Consensus statement on concussion in sport—The 5th International Conference on Concussion in Sport held in Berlin, October 2016. Br. J. Sports Med..

[6] Dompier T.P., Kerr Z.Y., Marshall S.W., Hainline B., Snook E.M., Hayden R., Simon J.E. (2015). Incidence of concussion during practice and games in youth, high school, and collegiate American football players. JAMA Pediatr..

[7] Zuckerman S.L., Kerr Z.Y., Yengo-Kahn A., Wasserman E., Covassin T., Solomon G.S. (2015). Epidemiology of sports-related concussion in NCAA athletes from 2009–2010 to 2013–2014: Incidence, recurrence, and mechanisms. Am. J. Sports Med..

[8] Gardner A.J., Iverson G.L., Williams W.H., Baker S., Stanwell P. (2014). A systematic review and meta-analysis of concussion in Rugby Union. Sports Med..

[9] Weber A.E., Trasolini N.A., Bolia I.K., Rosario S., Prodromo J.P., Hill C., Romano R., Liu C.Y., Tibone J.E., Gamradt S.C. (2020). Epidemiologic assessment of concussions in an NCAA Division I women’s soccer team. Orthop. J. Sports Med..

[10] Wasserman E.B., Kerr Z.Y., Zuckerman S.L., Covassin T. (2016). Epidemiology of sports-related concussions in National Collegiate Athletic Association athletes from 2009–2010 to 2013–2014: Symptom prevalence, symptom resolution time, and return-to-play time. Am. J. Sports Med..

[11] Leddy J.J., Burma J.S., Toomey C.M., Hayden A., Davis G.A., Babl F.E., Gagnon I., Giza C.C., Kurowski B.G., Silverberg N.D. (2023). Rest and exercise early after sport-related concussion: A systematic review and meta-analysis. Br. J. Sports Med..

[12] Hobbs J.G., Young J.S., Bailes J.E. (2016). Sports-related concussions: Diagnosis, complications, and current management strategies. Neurosurg. Focus.

[13] Snyder A.R., Greif S.M., Clugston J.R., FitzGerald D.B., Yarrow J.F., Babikian T., Giza C.C., Thompson F.J., Bauer R.M. (2021). The effect of aerobic exercise on concussion recovery: A pilot clinical trial. J. Int. Neuropsychol. Soc..

[14] Jo J., Williams K.L., Hill T.M., Perry G.M., Prosak O.L., Amedy A., Anesi T.J., Terry D.P., Zuckerman S.L. (2023). Return-to-learn after sport-related concussion: Does school level matter?. J. Neurosurg. Pediatr..

[15] Bahr R., Clarsen B., Derman W., Dvorak J., Emery C.A., Finch C.F., Hägglund M., Junge A., Kemp S., Khan K.M. (2020). International Olympic Committee consensus statement: Methods for recording and reporting of epidemiological data on injury and illness in sport 2020 (including STROBE Extension for Sport Injury and Illness Surveillance (STROBE-SIIS)). Br. J. Sports Med..

[16] Halonen J.I., Erhola M., Furman E., Haahtela T., Jousilahti P., Barouki R., Bergman Å., Billo N.E., Fuller R., Haines A. (2020). The Helsinki Declaration 2020: Europe that protects. Lancet Planet Health.

[17] Sunagawa N. (2022). Recommended methods for sports injury and illness surveillance. Japanese Society of Clinical Sports Medicine and Japanese Society for Athletic Training Consensus Document. Jpn. J. Athl. Train.

[18] Kimura T., Mącznik A.K., Kinoda A., Yamada Y., Muramoto Y., Katsumata Y., Sato K. (2023). Prevalence of and factors associated with sports injuries in 11,000 Japanese collegiate athletes. Sports.

[19] Abrahams S., Fie S.M., Patricios J., Posthumus M., September A.V. (2014). Risk factors for sports concussion: An evidence-based systematic review. Br. J. Sports Med..

[20] Tanaka S., Sagisaka R., Sone E., Tanaka H. (2023). Sport level and sex differences in sport-related concussion among Japanese collegiate athletes: Epidemiology, knowledge, reporting behaviors, and reported symptoms. Sports Med. Health Sci..

[21] Ianof J.N., Freire F.R., Calado V.T.G., Lacerda J.R., Coelho F., Veitzman S., Schmidt M.T., Machado S., Velasques B., Ribeiro P. (2014). Sport-related concussions. Dement. Neuropsychol..

[22] Tashima C., Otomo M., Hosokawa Y. (2024). History, Knowledge, and Education of Sport-Related Concussion Among College Athletes in Japan. J. Athl. Train..

[23] Van Pelt K.L., Allred D., Cameron K.L., Campbell D.E., D’Lauro C.J., He X., Houston M.N., Johnson B.R., Kelly T.F., McGinty G. (2019). A cohort study to identify and evaluate concussion risk factors across multiple injury settings: Findings from the CARE Consortium. Inj. Epidemiol..

[24] Eliason P.H., Galarneau J.M., Kolstad A.T., Pankow M.P., West S.W., Bailey S., Miutz L., Black A.M., Broglio S.P., Davis G.A. (2023). Prevention strategies and modifiable risk factors for sport-related concussions and head impacts: A systematic review and meta-analysis. Br. J. Sports Med..

[25] Trexler E.T., Smith-Ryan A.E., Blue M.N.M., Schumacher R.M., Mayhew J.L., Mann J.B., Ivey P.A., Hirsch K.R., Mock M.G. (2017). Fat-free mass index in NCAA division I and II collegiate American football players. J. Strength Cond. Res..

[26] McHugh C., Hind K., O’halloran A., Davey D., Farrell G., Wilson F. (2021). Body mass and body composition changes over 7 years in a male professional rugby union team. Int. J. Sports Med..

[27] Chatha K., Pruis T., Peaguda C.F., Guo E., Koen S., Malone D., Sabesan V. (2020). Concussions in soccer: An epidemiological analysis in the pediatric population. Orthop. J. Sports Med..

[28] Vomer R., Johnston K., Lau B.C., Bytomski J. (2022). Brain health considerations in the modern lacrosse athlete. J. Cartil. Jt. Preserv..

[29] Williams K., Zeoli T., Allen J.H., Jo J., Yengo-Kahn A.M., Terry D.P., Zuckerman S.L. (2024). Risk of two sport-related concussions in the same year: Is the second concussion worse?. Clin. J. Sport Med..

[30] Broglio S.P., Cantu R.C., Gioia G.A., Guskiewicz K.M., Kutcher J., Palm M., Valovich McLeod T.C., National Athletic Trainer’s Association (2014). National Athletic Trainers’ Association position statement: Management of sport concussion. J. Athl. Train..

[31] Thomas D.G., Apps J.N., Hoffmann R.G., McCrea M., Hammeke T. (2015). Benefits of strict rest after acute concussion: A randomized controlled trial. Pediatrics.

[32] Wang Y., Nelson L.D., LaRoche A.A., Pfaller A.Y., Nencka A.S., Koch K.M., McCrea M.A. (2016). Cerebral blood flow alterations in acute sport-related concussion. J. Neurotrauma.

[33] Leddy J.J., Wilber C.G., Willer B.S. (2018). Active recovery from concussion. Curr. Opin. Neurol..

[34] Dick R., Agel J., Marshall S.W. (2007). National Collegiate Athletic Association Injury Surveillance System commentaries: Introduction and methods. J. Athl. Train..

