# Supplementary material for: A One-Year Retrospective Study of the Occurrence of Sport-Related Concussions in Japanese University Sports: Characteristics of Athletes with Multiple Occurrences
Source: Sports (Basel). 2026 Apr 28;14(5):175. doi: 10.3390/sports14050175 (PMC13210862; doi:10.3390/sports14050175)
Supplement: Supplementary file 1 [file sports-14-00175-s001.zip › sports-4210371-supplementary.pdf]

Table S1. Comparison of physical characteristics between athletes with and without sport-related concussion by sport.

	SRC<br>(+)	SRC<br>(-)
Rugby Football		
Height (cm)	174.5<br>(5.9)	173.2<br>(6.7)
Mass (kg)	88.2<br>(12.6)	84.5<br>(13.6)
BMI (kg/m2)	**28.9***<br>**(3.3)**	28.1<br>(3.6)
American Football		
Height (cm)	172.6<br>(5.5)	173.5<br>(6.0)
Mass (kg)	84.1<br>(13.7)	81.4<br>(13.4)
BMI (kg/m2)	**28.2***<br>**(4.0)**	27.0<br>(3.9)
Lacrosse		
Height (cm)	165.6<br>(8.6)	164.6<br>(8.5)
Mass (kg)	60.1<br>(10.8)	59.0<br>(9.7)
BMI (kg/m2)	21.8<br>(2.2)	21.7<br>(2.2)
Soccer		
Height (cm)	172.5<br>(4.1)	170.6<br>(7.7)

Mass (kg)	66.4<br>(8.1)	64.0<br>(8.0)
BMI (kg/m2)	22.2<br>(2.1)	21.9<br>(1.5)

＊$p < 0.05$; significant differences between conditions with and without concussion.

SRC；Sport related concussion, BMI; Body mass index
